# What Makes the Time Tradeoff Tick? A Sociopsychological Explanation

**DOI:** 10.1177/0272989X241286477

**Published:** 2024-10-15

**Authors:** Peep F. M. Stalmeier, Bram Roudijk

**Affiliations:** Radboud Institute for Health Sciences, Nijmegen, the Netherlands; Radboud Institute for Health Sciences, Nijmegen, the Netherlands; EuroQol Research Foundation, Rotterdam, the Netherlands

**Keywords:** cultural values, death thoughts, euthanasia, religion, self-esteem, socioeconomic status, subjective life expectancy, terror management theory, time tradeoff, utilities

## Abstract

**Background:**

A theoretical interpretation of factors influencing time tradeoff (TTO) scores is lacking. In this conceptual study, we use a sociopsychological theory, terror management theory (TMT), to explain how death thoughts may play a role in the TTO method. TMT describes how respondents suppress death thoughts by invoking psychological defenses, such as self-esteem, and by bolstering cultural values.

**Research Question:**

What is the relation between TMT and TTO scores?

**Methods:**

A framework is developed to link TMT to TTO scores. Predictions of the framework pertain to the directionality of relations between characteristics (e.g., being religious) affecting TTO scores. These predictions are then tested against the findings in the literature.

**Results:**

The value “prolonging life” can be used as a linking pin between TTO and TMT as it is relevant for both TMT and TTO. It is argued that the value “prolonging life” is related to TTO scores but also to TMT defense strengths. Thus, TMT defense strengths become associated with trading. Directionality predictions of the framework were confirmed in 34 out of 39 retrospective tests (*P* < 0.001).

**Conclusion:**

Directionalities of relations between TTO scores and socioeconomic characteristics, euthanasia, subjective life expectancy, and religion were explained in terms of TMT defense strengths. Our framework offers a theory-based and parsimonious framework to think about characteristics influencing TTO scores.

**Highlights:**

- Terror management theory (TMT) is a sociopsychological theory about death thoughts.
- Several factors are known to influence TTO scores.
- A new framework applies TMT to TTO scores to interpret such factors.
- Our framework is mostly of importance to health economists studying the TTO.

## Background

In health economics, several methods are used to measure the value of health, resulting in a single index measure. Such health values or health utilities usually lie between 0 (being dead) and 1 (perfect health) and can be used for health care decision making.^1^ In the time tradeoff method (TTO), widely used to assess values for health, respondents sacrifice hypothetical life-years in good health to avoid living in a lesser health state.^2,3^ As an example, consider the state “being in a wheelchair.” A respondent willing to sacrifice or trade 3 y in good health is indifferent between 7 y in good health and 10 y in a wheelchair. Then, under certain assumptions, the TTO value of living in a wheelchair equals 0.7.^4,5^ For more severe health states, sacrificing more life-years would be expected, corresponding with lower TTO scores. We are interested in the interpretation of factors influencing TTO scores. Specifically, these factors lie within the respondent. External factors such as the health state descriptions, group exercises, or online nature of the task, are not considered.

The TTO method and other valuation methods entail comparisons with dead.^2,6–8^ Health states are described, for instance, as 10 y followed by dead. Health states can be valued as better or worse than dead. The trading process itself involves comparing states with longer or shorter survival. In line with this, qualitative studies report many verbal utterances involving (comparisons with) dead while respondents perform the task. For instance, respondents believe that some health states could be worse than dead.^8^ Other illustrative utterances are: “I do not know what happens after I am dead” or “when I’m dead, whether it is dead, or near dead, after death or before death, to me it’s just dead.”^8^ In another study, respondents stated that “at the moment I wouldn’t want to say that I want to die immediately but that might be because I can’t believe or imagine it,” or yet another example: “If you’ve got no one to support you through any of those things I can completely understand why you would want to die.”^9^

This suggests that death thoughts enter the mind while doing such health valuation tasks.^8,9^ In the health economics literature, theorizing about the psychological consequences of death-related thoughts is absent. Certainly, theories on judgment and decision making address effects such as framing, sensitivity to risk, and outcomes for standard gambles, and they address measurement properties for the TTO such as constant proportional tradeoff or theorize about time preferences.^4,10–18^ However, these models usually remain in the domain of the variables presented in the stimuli (e.g., health state descriptions, monetary outcomes, risks, or durations). These theories do not address the psychological consequences of undergoing death-related thoughts in the valuation process.

Terror management theory (TMT), however, deals specifically with death thoughts.^19–22^ It is a sociopsychological theory describing human defenses against threats to self-preservation and death anxiety, caused by death-related thoughts. TMT uses concepts outside the scope of theories on judgment and decision making. Our proposition is that the TTO task engenders thoughts about death, and given that TMT is a theory about defenses against death thoughts, it is worthwhile to connect the TTO method with TMT.

TMT is hence proposed to improve our understanding of characteristics and values influencing TTO scores. This endeavor is relevant as not much is known about factors influencing TTO scores and it is often unclear how to interpret them.^23–26^ Please note that it is not our aim to rule out other explanations. Our aim is to show that TMT and a single working hypothesis are able to explain the observational findings. The aims of this conceptual study are therefore 1) to develop a framework to link TMT and TTO and 2) to test hypotheses derived from this framework. The link between TTO and TMT is not proven but works well for predictive purposes. Below, the next section presents a cursory account of TMT. The third section proposes a process linking TTO with TMT concepts. The fourth section presents the results of retrospective hypothesis testing. The fifth section contains the discussion. Hereafter, the term *TTO score* is often replaced by the inversely related “number of years traded.”

## A Cursory Account of TMT

### Introducing TMT

TMT is a sociopsychological theory describing defenses against anxieties raised by knowing our mortality. According to TMT, self-preservation and cognition relate to our reactions toward death. Cognition renders a conscious awareness of a future death and creates terror.^19,27^ In typical TMT studies, a death reminder, which is mortality salient (MS) stimulus, creates death thoughts. Examples of death reminders are thinking about one’s own death or cancer.^20^

TMT proposes that humans keep terror under control through faith in their cultural worldview (CWV).^28^ A CWV is a way of thinking about the world and may entail normative postulates, values, and ethics.^22,29,30^ The CWV is individual and unique, although shaped by the overarching culture individuals live in.^29,31,32^ Transcending death may be “either literally (through beliefs in an afterlife), or symbolically (by leaving a lasting impact on the world though group identification, relationships, children, or other life achievements).”^28^ Anxiety reduction through defending the CWV can be loosely summarized as “the individual dies, but culture persists.” CWV defense indeed reduces the accessibility of unconscious death thoughts.^33^ For instance, religion entails a promise of a life after death, and after MS, religious faith was strengthened.^27,32^

Two personality variables, self-esteem and secure attachment, moderate the effects of MS. In experimental studies, CWV defense together with self-esteem and secure attachment were found to reduce unconscious death thoughts.^33^ Self-esteem develops because children depend on their parents to sooth their anxieties.^22,28,33,34^ By living up to their parents’ standards, children feel worthy, that is they feel self-esteem, approval, and protection. This makes self-esteem an anxiety buffer. At older age, the basis of self-esteem transfers to living up to societal standards (e.g., to one’s nation or religion).^28^ This process occurs in every culture. Secure attachment also develops in early childhood. Securely attached people score low in relationship-related anxiety and avoidance.^28,33^ Secure attachment is intertwined with self-esteem, and both have been shown to reduce death thoughts.

The above 3 defenses, CWV defense, self-esteem, and secure attachment, are referred to as the 3 distal defenses because they function when death thoughts are unconscious.^35^ When thoughts of death are consciously present, the so-called conscious proximal defense comes into play. “Proximal defense . . . functions to either push death thoughts out of consciousness or to push the threat of death into the distant future.”^35,36^ Proximal defenses may consist of distraction (listening to music) or may consist of logical reasoning (e.g., by reminding yourself you have a healthy lifestyle or that you have quit smoking).^35^ The conscious proximal defense, in the sense of pushing death to the future, is central in our reasoning.^34^

A large number of studies supporting TMT are described in a meta-analysis by Burke et al.^37^ Experimental setups used in TMT are described in Hayes et al.^20^ An example of a TMT experiment is now described.

### An Example of a TMT Study

TMT was used to predict the number of goals for an upcoming Dutch–German soccer match. As the national team can be seen as part of the Dutch identity, the prediction was that those reminded of death would predict more goals for the Netherlands.^38^ It was found that without a death reminder, respondents predicted that the match would end more or less in a draw. However, after a death reminder, the number of goals predicted in favor of the Dutch team increased from 1.1 to 1.6, allowing a clear victory for the Dutch team. This highlights that worldview values, in this case the cultural value of the national soccer team, were defended after a reminder of death.

## A Framework Linking TMT to TTO

In this section, a framework linking TMT and TTO scores is described. Importantly, this framework is formulated in terms of defense strengths and not in terms of death thoughts. Whereas TMT is a theory confirmed by randomized experimental designs, the framework is based on reasoning about associations and, later on, tested against observational findings in the TTO literature.

### A Higher Value of the Value “Prolonging Life” Is Related to Less Trading

In 5 independent study centers, it was found that respondents more strongly endorsing the value “prolonging life” traded fewer life-years.^23–25,39–42^ Kirsch and McGuire^39^ found that respondents who expressed a strong preference for staying alive “at all costs” were reluctant to tradeoff any years. Van Nooten et al. asked respondents, “If, due to some illness, you had to choose between a shorter life in good health and a longer life in poorer health, what would you choose at this moment?” Individuals preferring a longer life traded fewer years.^23–25^ In a similar vein, 3 separate research groups found that respondents preferring “length of life,” as opposed to quality of life, traded fewer life-years.^40–42^

The value “prolonging life” is used to link TMT and TTO scores. It may seem that alternative values would be equally plausible (e.g., “embracing death” or “life is enough”). However, for “prolonging life,” there is convincing evidence from 5 research groups relating this value to TTO scores. For the alternative values, such evidence is lacking as far as we know. Also, the use of the value “prolonging life” makes the reasoning in the following paragraph more natural. Therefore, our choice for the value “prolonging life” is both evidence based and practical.

### The Value “Prolonging Life” Is Related to the Proximal Defense

In this section, the proximal defense and TTO are linked. This link has never been proven as such, but it is a plausible link, which can be tested empirically. The link is used as a working hypothesis needed to formulate the hypotheses tested later on. As described, the proximal defense pushes death into the distant future or uses distraction, to reduce death thoughts. In terms of values, a stronger proximal defense amounts to a higher value of “prolonging life.”^36^ Similarly, in TTO, death is pushed to the future when fewer years are traded. For instance, to avoid 10 y in a severe health state, one respondent sacrifices 6 y in full health. A second respondent may sacrifice only 4 y. All other things being equal, the second respondent pushes death 2 y further into the future. By the previous section, this amounts to a higher value for life duration or “prolonging life” for the second respondent, who trades fewer years.^23–25,39–42^ Because both the proximal defense and trading behavior relate to “prolongation of life,” an association is expected between the proximal defense strength and the number of years traded. This relation is inverse because in TMT, a stronger proximal defense strength will push death further to the future (more “prolongation of life”); then, according to the previous section, less trading occurs when “prolonging” life is enhanced. We assume that this inverse relation holds within respondents to generate our hypotheses for factors influencing TTO.

### Alignment of the Defense Mechanisms

There are 4 defenses to reduce conscious and unconscious death thoughts, namely, the 3 unconscious distal defenses, self-esteem, secure attachment, worldview defense, and the conscious proximal defense. Threats to one component of the defense system result in defensive activation of other components.^33^ After a death threat, initially the proximal defense reduces death thoughts.^34^ Later on, the distal defenses take over to reduce remaining unconscious death thoughts.^27,32,33,36,43,44^ As elevating any defense reduces death thoughts, the 4 defenses work together or are aligned to reduce death thoughts. Therefore, any increase in unconscious defense strength strengthens the proximal defense. This leads to increased importance of the value “prolonging life,” which in turn affects trading in TTO, (section 2.1). This process is again assumed to take place within respondents. In short, all TMT defenses are now linked to trading years. Please note that the framework is formulated terms of defense strengths.

### Testable Hypotheses

Since proximal and distal defenses are aligned and associated with the “prolonging life” value, a prediction can be made about the sign, or directionality, of the association between TMT defense strengths and TTO scores. The sign depends on characteristics of the respondent and whether or not these characteristics lead to stronger or weaker defenses. For instance, the cultural value “desire for offspring” was found to be strengthened after mortality salience (MS), indicating a strengthened CWV defense.^37^ As defenses are aligned, the proximal defense is strengthened as well, and in turn the value “prolonging life” is increased, resulting in less trading. Hence, those more strongly endorsing “desire for offspring” are hypothesized to trade fewer years. Such hypotheses are evaluated retrospectively in section 3. Please note that whether or not a characteristic strengthens or weakens a defense is interpreted strictly within the realm of TMT.

### Summary of the Framework

**Figure 1:**
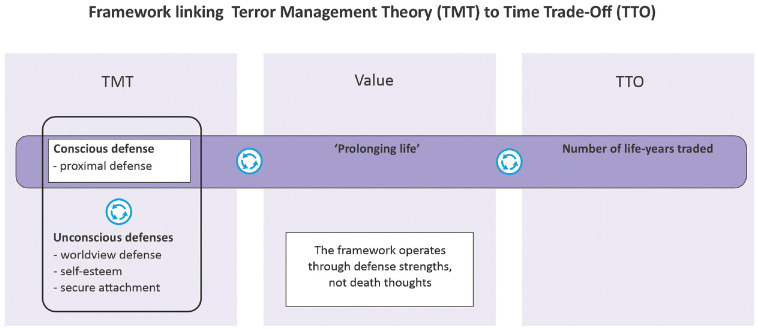
Framework linking terror management theory (TMT) to time tradeoff (TTO).

The above reasoning is summarized in Figure 1. The value “prolonging life” is an intermediary value linking TTO and TMT (Figure 1, middle panel). The value “prolonging life” affects trading in the TTO (Figure 1, right 2 panels) and is associated with the proximal defense (Figure 1, left 2 panels). All TMT defenses, including the proximal defense, work together and are associated (Figure 1, left panel).

The key message is that stronger TMT defenses lead to less trading. The directionality of any hypotheses is then related to how variables relate with the value “prolonging life.” For example, a stronger conscious proximal defense is associated with a stronger “prolonging life” value, and less trading is expected. Stronger unconscious defenses, that is a stronger CWD, higher self-esteem, and higher secure attachment, are positively associated with a stronger proximal defense, and again less trading is expected. To conclude, stronger defenses (left panel) lead to a higher “prolonging life” value (middle panel) and hence to less trading (right panel).

## Retrospective Testing

### Literature Search

We conducted a literature review to identify papers using the TTO or lead-time tradeoff methods and reporting on characteristics that significantly affect the TTO. First, the papers by van Nooten and Devlin served to generate relevant characteristics influencing the TTO.^23,26^ Second, PubMed was searched for additional studies related to these characteristics using search terms such as [TTO or “time trade-off” or “time tradeoff” or “time trade off”] combined with, for example, [“education” or “having a degree”]. Third, the first authors’ relevant archives were searched. Fourth, Medline (Ovid) weekly literature alerts, in the period August 2020 to March 2022, were searched; the alerts involved (patient) preferences and utilities (see the appendix).^45^ Relevant papers were selected, and their tables were thoroughly examined for characteristics influencing the TTO. Results of multivariate analyses were used whenever present.

Our literature search diverges from the conventional meta-analysis or systematic review question such as, “What is the effect of variable A on outcome B?” Instead, the aim of the search is to identify studies with significant characteristics, whose directionality can be tested against the framework’s predictions.

### Analyses

Characteristics were scored on whether or not they confirmed the directionality hypotheses of the framework regarding more or less trading. These hypotheses were generated as explained in the Summary of the Framework. Studies with several significant sociodemographic characteristics were conservatively counted as disconfirming if at least 1 characteristic significantly disconfirmed a hypothesis.

## Results

The first 3 search strategies yielded 27 papers. The Medline (Ovid) search yielded 1,849 papers of which 10 papers were not identified earlier.^41,46–54^ For the first 3 search strategies, no systematic records were kept. The papers uncovered a number of characteristics affecting the TTO scores: sociodemographic, attitudes toward euthanasia, subjective life expectancy, and religion. Results of the hypotheses testing are summarized in Table 1.

**Table 1:** Results of Retrospective Hypothesis Testing: Number of Predictions of Our Framework Agreeing with the Direction of Significant Characteristics as Reported in Independent Studies

Characteristic	Number of Hypotheses Confirmed
Socioeconomic status characteristics	19 out of 23 studies
Euthanasia	3 out of 3 studies
Subjective life expectancy	3 out of 3 studies
Being religious	7 out of 8 studies

A total of 32 of 37 hypotheses were confirmed, which is significant in a binomial test with 50% (random directionality agreement) as the null hypothesis (*P* < 0.001). Studies with multiple significant sociodemographic characteristics were counted as disconfirming if at least 1 characteristic was in the wrong direction.

### TMT and Socioeconomic Characteristics

We found 23 studies examining socioeconomic characteristics. In 19 studies, being married, being in work, having children, longer education, having stable housing, were significantly related to trading fewer years,^21,23,26,41,42,46,49,50,52,54–63^ These studies were about self-experienced health or hypothetical states. Four studies, of which 2 were online studies, reported opposite results.^7,47,48,53^ The literature offers a variety of explanations for the observed significant associations. For instance, it is suggested that time perspective may differ others value their time more and therefore are less willing to trade time.^26^ One study mentioned family and witnessing child’s milestones as being important.^56^ Matza et al.^63^ suggested that parents may have increased altruism and a sense of responsibility for another individual’s well-being. One may also hold that “the economic cost of dying early” is higher for those better off. Such descriptive explanations are reasonable but not causal as they are not confirmed by experimental designs.

The framework uses the self-esteem defense to explain that elevated socioeconomic characteristics give rise to less trading. Our reasoning is that socioeconomic characteristics may reflect status, elevating self-esteem. Observational studies indeed found that self-esteem is positively related to elevated status.^64–67^ Hence, our framework predicts that those with elevated status, and correspondingly higher self-esteem, show less trading. This prediction is confirmed: for higher status characteristics, 19 of 23 studies found less trading. Another study showed that higher self-esteem was clearly associated with less trading in patients valuing their self-experienced health and suggests that for hypothetical health states, self-esteem can explain up to 5% the variance in trading.^68^ Please note that the framework offers a single explanation in terms of self-esteem, which is more parsimonious than the variety of explanations offered in the literature.

### TMT and Attitudes toward Euthanasia

Regarding euthanasia, 3 out of 3 studies independently reported significantly more trading among respondents favoring euthanasia or its legislation.^21,25,69^ One study proposed that “individuals’ acceptance of death and their attitudes towards euthanasia may be the underlying construct . . . .that underlies trading more time.” This study also suggested that “religious reasons could restrain respondents from directly stating that the target health state is worse than dead, or that other constructs such as religion, fear of death, etcetera are involved.”^69^ Another study mentioned the maxims of religious teaching, namely, that life has intrinsic value.^21^ This study noted the lack of causal explanations and the possibility of unobserved moderating characteristics.^21^ The third study did not offer an explanation.^25^

The explanation of the framework is that favoring euthanasia entails a reduction of the prospect of longevity. Since the proximal defense aims at prolonging longevity, favoring euthanasia goes against, and therefore weakens, the thrust of the proximal defense. As explained before, a weakened defense decreases the value “prolonging life” and more years will be traded for those favoring euthanasia, as was indeed found in 3 out of 3 studies.

### TMT and Subjective Life Expectancy

Regarding subjective life expectancy (SLE), 3 studies reported that respondents with higher SLE trade fewer years.^51,70,71^ These studies explained their findings in terms of prospect theory, namely that respondents trade fewer years when TTO durations are seen as losses relative to the SLE. These explanations are confined to the characteristics SLE and duration. One study attributed the finding to a biased estimate of life expectancy relative to life tables.

The explanation of the framework is that respondents with a higher SLE shift the prospect of death further into the future, similar to the aim of the proximal defense. Therefore, a higher SLE strengthens the proximal defense, and less trading is expected, as was indeed found in 3 out of 3 studies.

### TMT and Being Religious

Six studies found that religion or a belief in an afterlife decreased trading.^42,52,55,61,72,73^ One study mentioned that those with a belief in the afterlife were less likely to trade, which was then tautologically explained by “the religious oppose voluntarily foregoing life years,” or “the religious simply value longevity more than quality.” Mrus et al.^55^ also gave a tautological explanation, namely, that those “having a religious affiliation” traded fewer years, “implying that people with higher levels of spirituality may be less willing to trade time.” One study explained the impact of religion as an obligation to preserve a God-given gift of life.^52^ An online study found the opposite result.^7^

Several qualitative studies suggest that trading life-years reminded respondents of “being more religious” and “that health, life, and death are in God’s hands.”^74–76^ These qualitative findings support the explanations given above but also the following explanation of our framework.

In TMT, “belief life after death, heaven, or reincarnation” strengthens distal defenses because such beliefs bestow “literal immortality”. In addition, evidence from TMT experiments shows that “making respondents think of religion” strengthens the defenses.^32,77–79^ On these 2 accounts, less trading is expected as found in 6 out of 7 studies.

## Discussion

### Main Findings

This is a conceptual study. It deals with the question regarding how factors influence the number of years traded in the TTO. From the literature, it is clear that the TTO task induces thoughts about death. TMT is a sociopsychological theory about human defenses against death-related thoughts. This study attempts to tie TMT to the number of years traded in the TTO task. According to TMT, strengthening TMT defenses reduces the terror of death. A framework is proposed that links TMT defense strength, and not death thoughts, via the intermediary value “prolonging life” to trading life-years in the TTO task. Hypotheses were derived and confirmed in retrospective tests.

### Interpretation

Causality is the first issue to consider. Explanations in the literature are based on observational data so they cannot claim the highest level of causality, which depends on randomization. Our framework depends on TMT. Within TMT, the level of causality is reflected by randomized designs. Our own framework makes predictions in terms of strengthened or weakened defenses. These predictions were confirmed in retrospective tests, again using observational data. Therefore, the framework cannot claim the highest level of causality and is on the same causality level as other explanations in the literature.

Parsimony is the second issue. It is certainly not our aim to fault or to rule out other explanations. For instance, it seems plausible to give explanations such as 1) it is likely that having children is protective against the choice to give up life-years, or 2) that the religious trade fewer years, or 3) those favoring euthanasia give a lower value to bad states. Our explanations, however, are based on a single and reasonable working hypothesis, namely that the value ‘prolonging life’ is related to the proximal defense. Having a single explanation makes our approach parsimonious and therefore scientifically desirable.

Directionality is the third issue. Consider the finding that those with children trade fewer years, explained, for instance, out of a “desire to see milestones.” What would the explanation be if the findings would have been reversed (e.g., that those with children trade *more* years)? Then the explanation could be a desire not to be a burden on adult children. Thus, different explanations can be used to fit either directionality. More generally, explanations in the literature often merely reflect the experimental findings, for example, “those with children are less prone to trade years,” which is tautological. Only if a theory is used to predict directionality can such explanations be faulted. Please contrast this with our framework that yields specific predictions about the direction of the associations based on defense strengths.

One may be surprised that TMT, a theory dealing with the functioning of cultural values, is linked to the TTO, a stated preference-based method. However, in qualitative and quantitative studies using the TTO, respondents spontaneously mention values such as independence, dignity, effect on family and children, religious belief, preservation of life, the importance of hope, meaning in life, and many other values.^6,8,9,73–75,80–86^ Apparently, values matter in TTO as well.^87^

Can the framework be extended to other health measures such as the standard gamble or applied to any form of preference research that includes death as a potential outcome? According to Hayes, even asking for the value and meaning of life itself already induces death thoughts, so our framework may be relevant.^20,37,88^ Our framework could be considered to explain other findings. For instance, it was found that attitudes toward euthanasia affected both TTO and visual analog scale (VAS) values, but that the effect on VAS values was weaker. Similarly, self-esteem affected TTO values in self-experienced health but much less so for hypothetical health states.^68^

### Strengths

A framework linking TMT and TTO was developed. Literature findings regarding the years traded in the TTO task for socioeconomic status (SES) characteristics, euthanasia, SLE, and religiosity were explained. Further evidence supporting our framework comes from qualitative interviews, which report thoughts of death in the TTO and the mention of values in qualitative and quantitative studies on the TTO. Falsifiable prospective tests of our framework are formulated for future work.

### Limitations of the Framework Building

The main assumption is that the value “prolonging life” is related to the proximal defense. This assumption seems plausible, but more importantly, it is possible to test it by measuring the relation between the proximal defense strength and the value “prolonging life” (see future work). A limitation of the framework is that it is assumed to operate within respondents.

### Limitations of Retrospective Testing

In the retrospective hypothesis testing, confirmation bias is an issue. To the best of our knowledge, systematic bias was absent as no distinction was made between literature findings confirming or discrediting our hypotheses. Another source of confirmation bias is the interpretation within TMT of characteristics that significantly influence trading. These interpretations might have unintendedly been such as to fit to the TTO evidence. This was not the case as our framework was developed independently before the retrospective hypothesis testing was conceived. Also, the interpretations of significant characteristics within TMT are transparently presented above and open to scrutiny.

### Limitations of the TTO Data

The TTO literature search, although partly reproducible, was not systematic as this was outside the scope. Studies were selected if characteristics significantly affected the TTO. In practice, in some studies, particular socioeconomic characteristics were significant but not in other studies. Whether characteristics are significant depends on the study design (e.g., sample size, online versus face-to-face data collection, and the distribution of TTO scores). Cultural characteristics may play a role (e.g., being religious varies across countries).^24,73,89^ The result of this selection process is 37 studies with significant results. Some significant findings may have occurred by chance. Also, there is a lack of studies reporting on TTO determinants, either because few studies are done in this area or because negative results are underreported. We also found that significant results were not reported in abstracts, hampering the search process.

### Relevance

We proposed a framework linking TMT and TTO. The framework is capable of explaining the observational findings regarding characteristics affecting the TTO. There are many explanations in the literature, whereas our framework 1) provides a parsimonious explanation to account for the observation findings and, 2) predicts the direction of the associations. As mentioned before, it is certainly not our aim to fault or rule out existing explanations. Our framework is of primary importance to health economists studying the TTO as it offers another interpretation of factors influencing TTO scores. Further, clinical research has shown that fewer death thoughts are associated with better emotional well-being and that the latter leads to less aggressive treatment choices.^90–92^ Clinicians may ask patients about their life domains or life goals. This may strengthen their “self-related worldview,” thus reducing death thoughts.

### Areas for Future Research

First, unconscious thoughts about death can be measured with a simple word completion task, for instance, whether the incomplete word “coff.” is completed as “coffee” or “coffin.” These unconscious thoughts may be used to explore differences between TTO or VAS tasks.^25,31^ Second, investigations are planned to explore the role of proximal and distal defenses and the value “prolonging life” in relation to trading.^20,93^ Third, one may test the prediction that high self-esteem and securely attached respondents display less willingness to trade. Relevant questionnaires are available.^30,94,95^ Fourth, sociometer theory holds that belonging in the context of social groups gives rise to elevated self-esteem. Thus, for respondents with elevated belonging, less trading is expected.^96–99^ Participation in various groups (e.g., committees, kin relationships, or friendships) could be considered as a proxy for belonging. Questionaires for belonging exist.^100,101^ Fifth, considering that TMT focuses on values, it is expected that stronger associations with trading occur for values compared with SES characteristics (e.g., one might use the value “striving for education” instead of the SES question “education” or “striving for close relationships” instead of “being married”). Sixth, the connection between values and TTO scores may be enhanced by studying trading for self-experienced health as this may evoke thoughts such as “who am I” or “what is important to me,” potentially strengthening associations with trading due to the heightened significance of personal values.^68,102^

## Supplemental Material

sj-docx-1-mdm-10.1177_0272989X241286477 – Supplemental material for What Makes the Time Tradeoff Tick? A Sociopsychological Explanation Supplemental material, sj-docx-1-mdm-10.1177_0272989X241286477 for What Makes the Time Tradeoff Tick? A Sociopsychological Explanation by Peep F. M. Stalmeier and Bram Roudijk in Medical Decision Making
