# Supplementary material for: What Makes the Time Tradeoff Tick? A Sociopsychological Explanation
Source: Med Decis Making. 2024 Oct 15;44(8):974–85. doi: 10.1177/0272989X241286477 (PMC11542326; doi:10.1177/0272989X241286477)
Supplement: sj-docx-1-mdm-10.1177_0272989X241286477 – Supplemental material for What Makes the Time Tradeoff Tick? A Sociopsychological Explanation [file sj-docx-1-mdm-10.1177_0272989X241286477.docx]

**Appendix:** Medline (Ovid) search

((tto not tea) or time trade* or "explode Quality-Adjusted-Life Years" or "explode Gambling").mp. or (gamble.mp. not gamble.in,au.) or ("explode Gambling-" or euroqol or health utilit* index).mp. or (hui.mp. not hui.au,jn.) or (treatment adj preference).mp.

not editorial.pt. not comment.pt. not letter.pt. not chinese.lg. not pest.jn.

limit 1 to dt=20200801-20220228
